# Supplementary material for: NPC1 Confers Metabolic Flexibility in Triple Negative Breast Cancer
Source: Cancers (Basel). 2022 Jul 21;14(14):3543. doi: 10.3390/cancers14143543 (PMC9319388; doi:10.3390/cancers14143543)
Supplement: Supplementary file 1 [file cancers-14-03543-s001.zip › Supplemental Data/annotated westerns.pdf]

Blot 1B

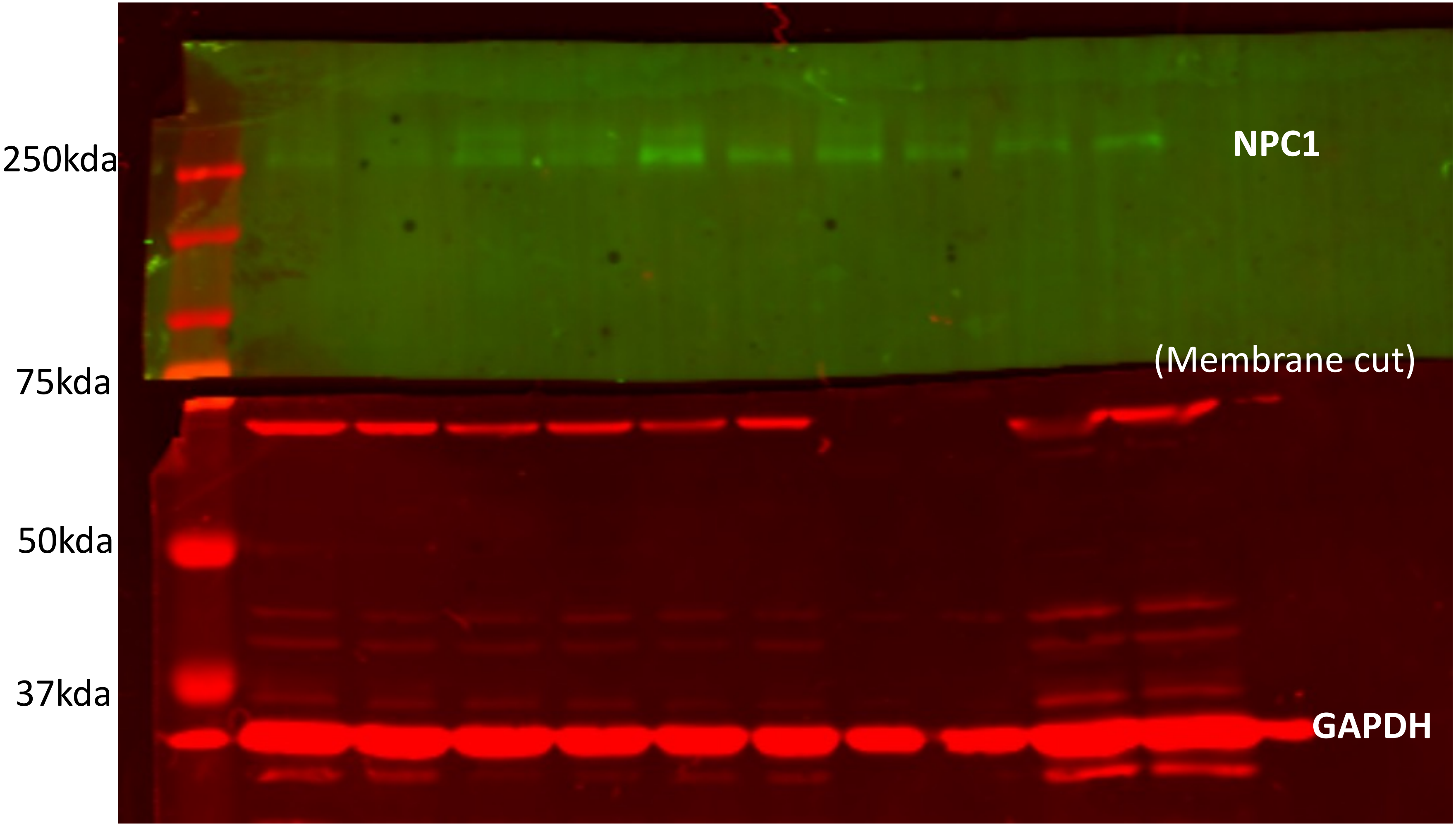

Blot 1F

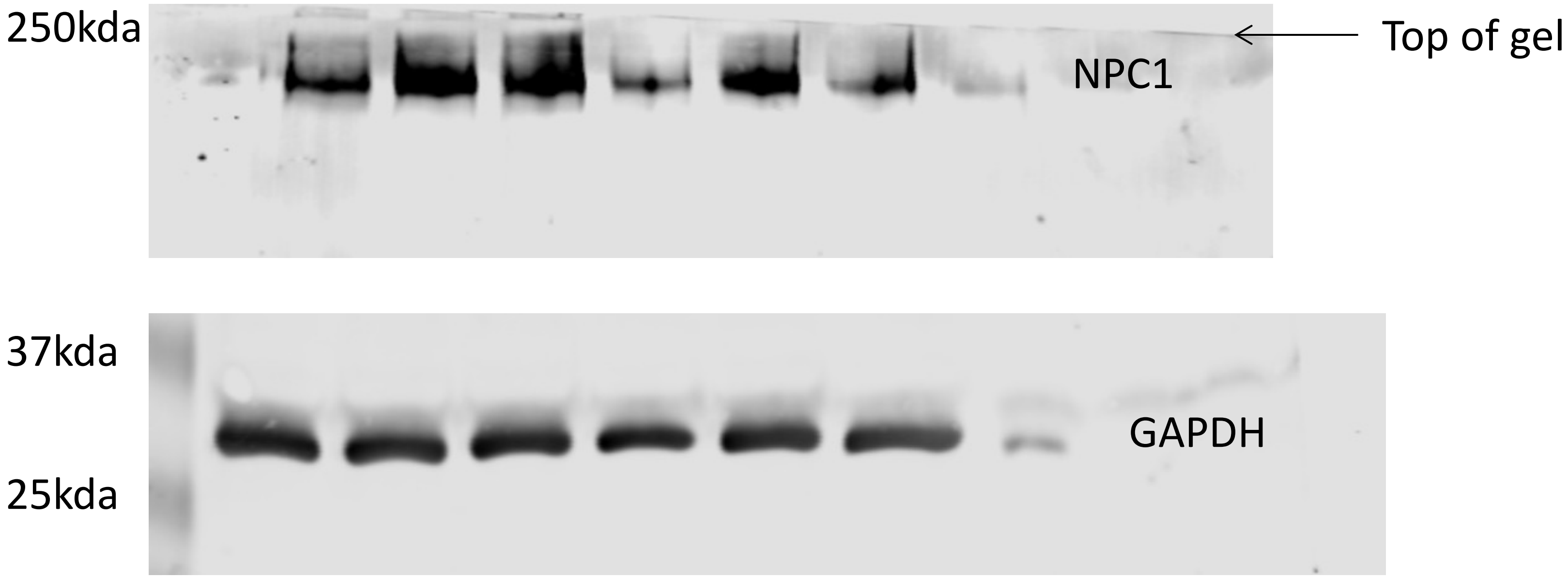

Blot 1H

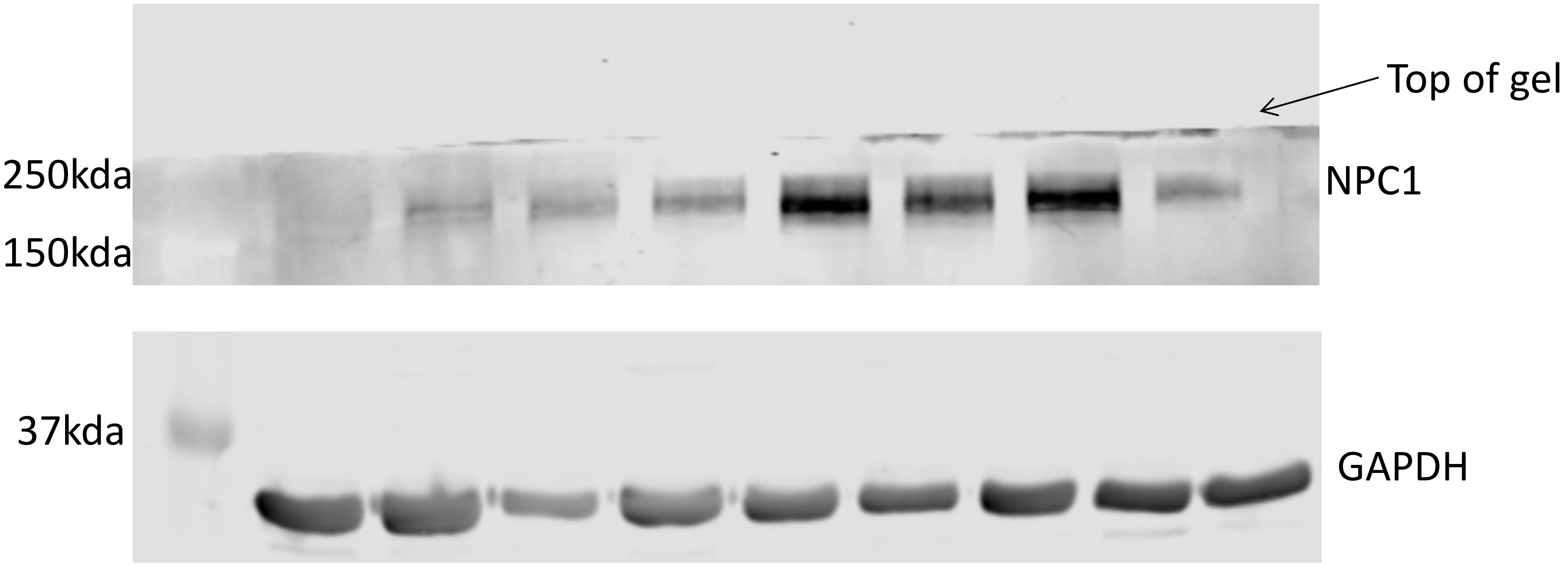

Blot 2A

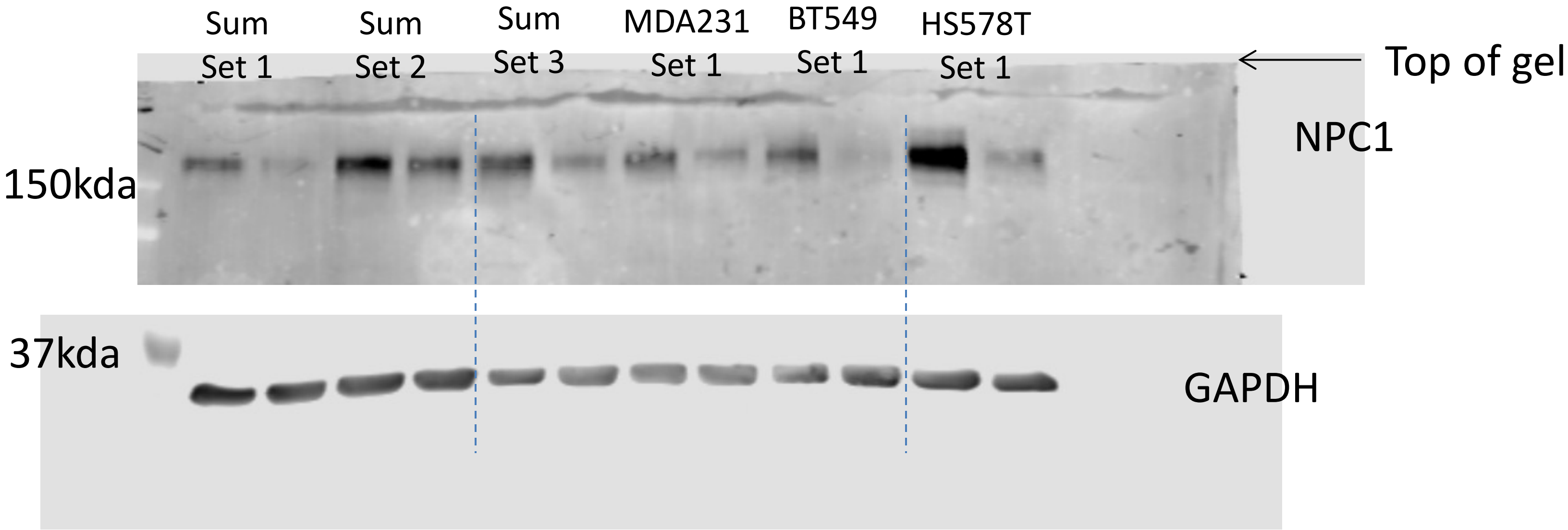

Sum Set 1
Sum Set 2
Sum Set 3
MDA231 Set 1
BT549 Set 1
HS578T Set 1
Top of gel
NPC1
150kda
37kda
GAPDH

Blot 3C

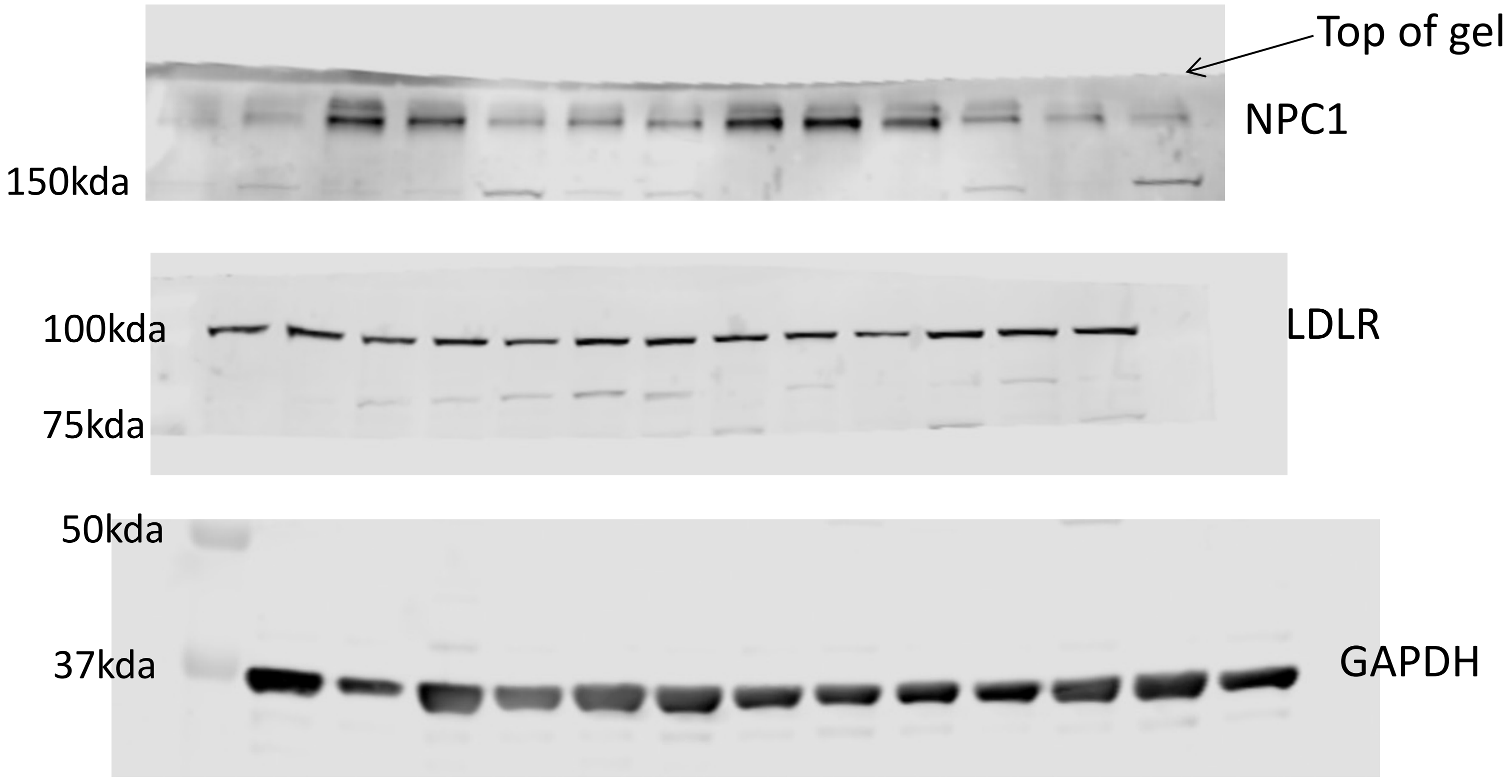

Blot 3C

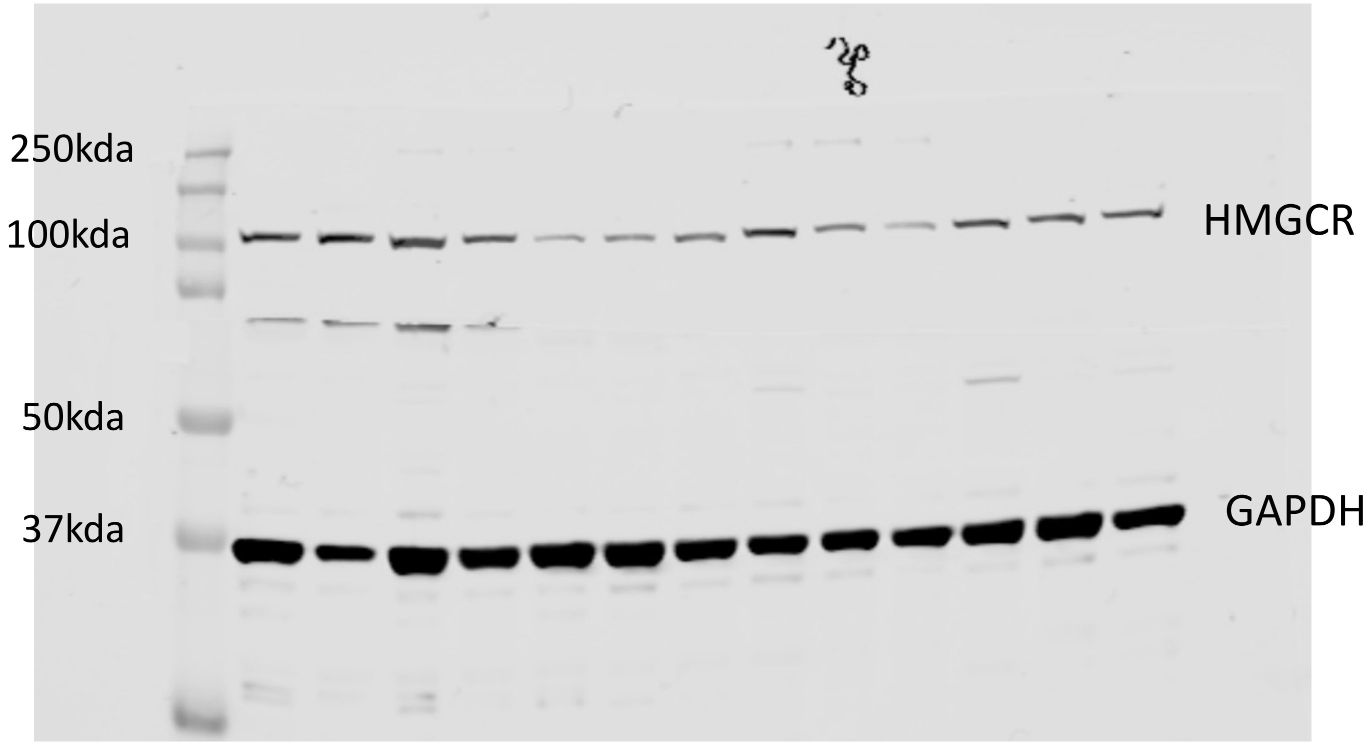

Blot 3E

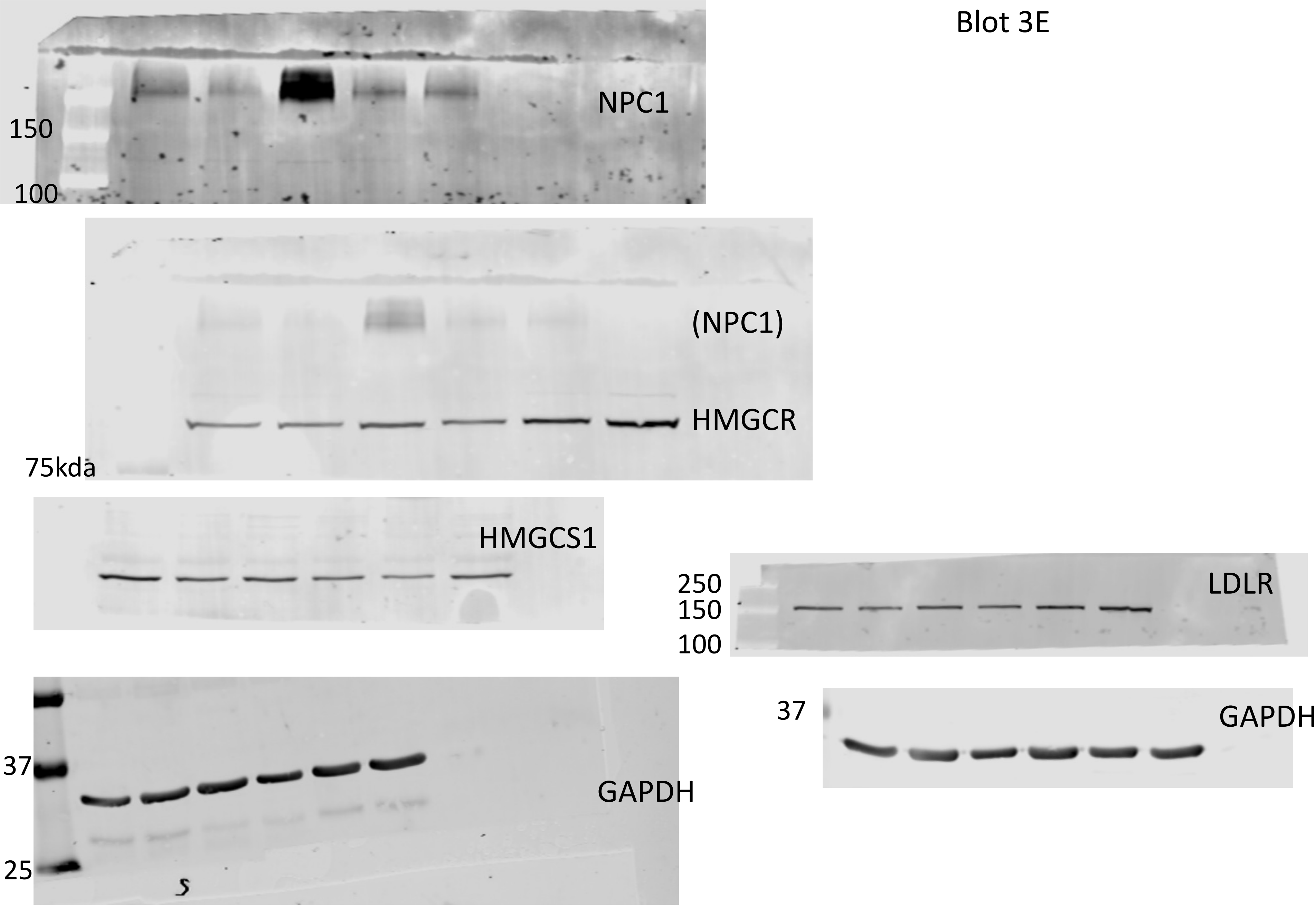
NPC1
150
100
(NPC1)
HMGCR
75kda
HMGCS1
LDLR
250
150
100
37
25
GAPDH
37
GAPDH

Blot 5A

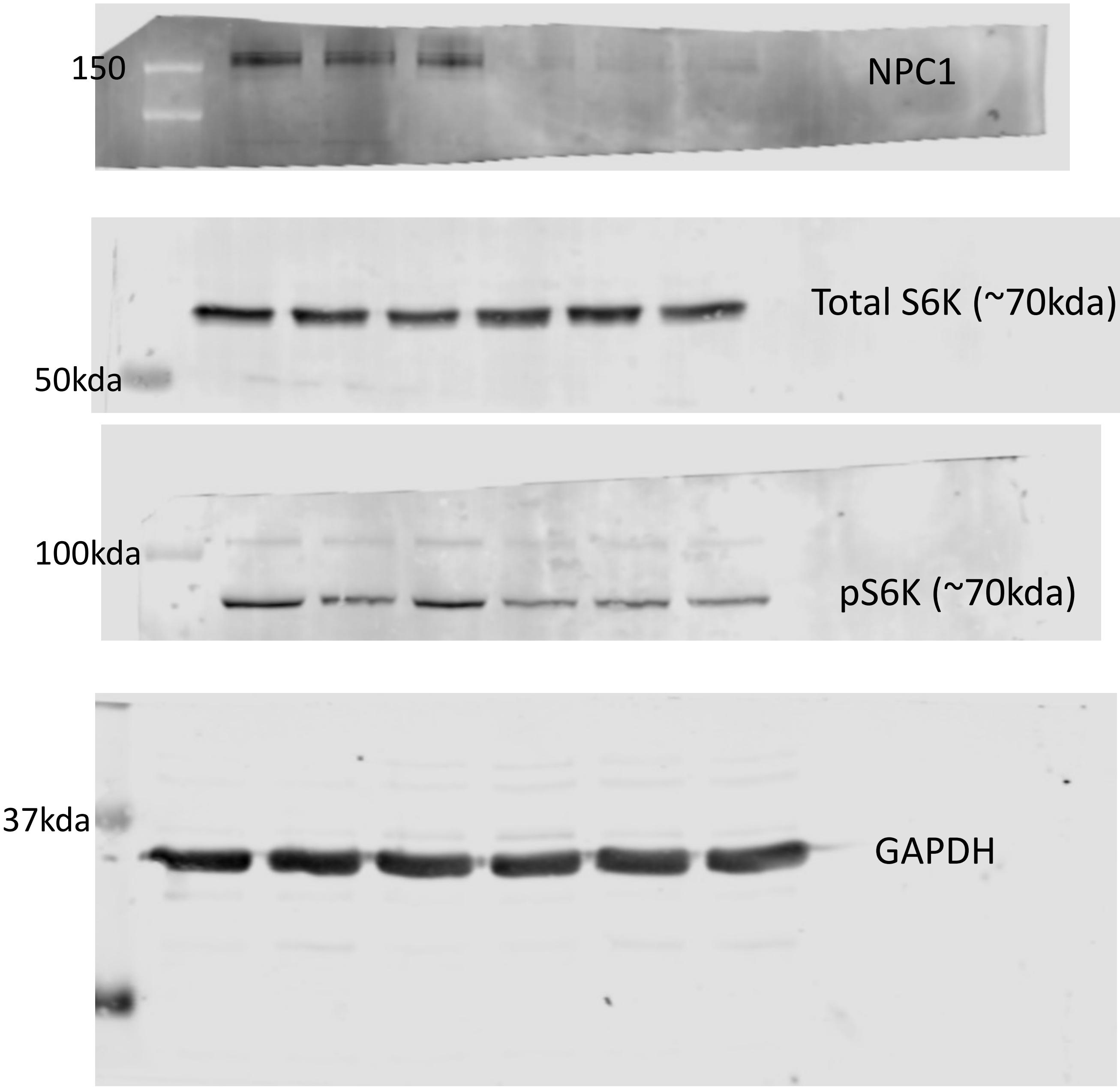

150
NPC1
Total S6K (~70kda)
50kda
100kda
pS6K (~70kda)
37kda
GAPDH

Blot 5B (1 of 2)

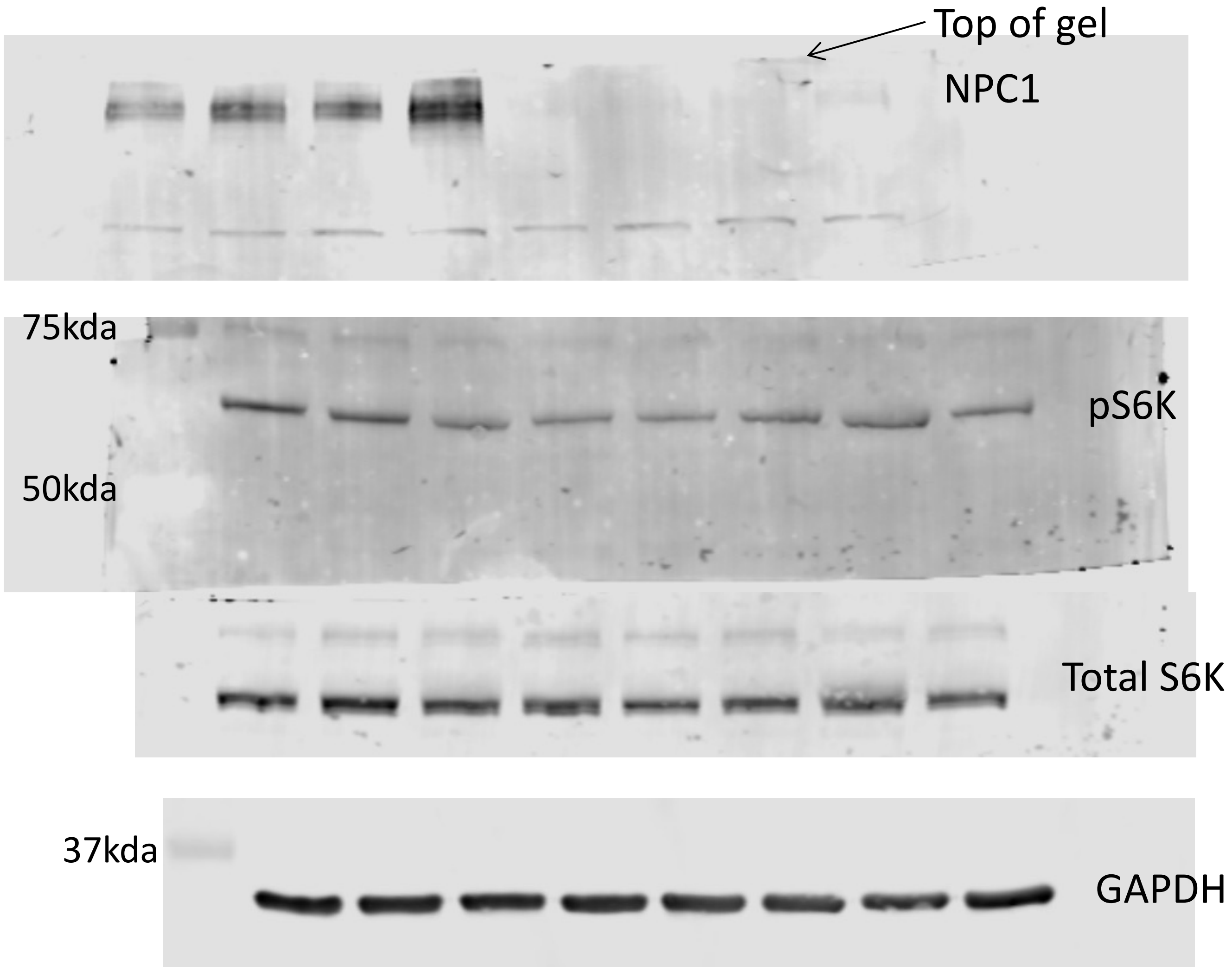

Blot 5B (2 of 2)

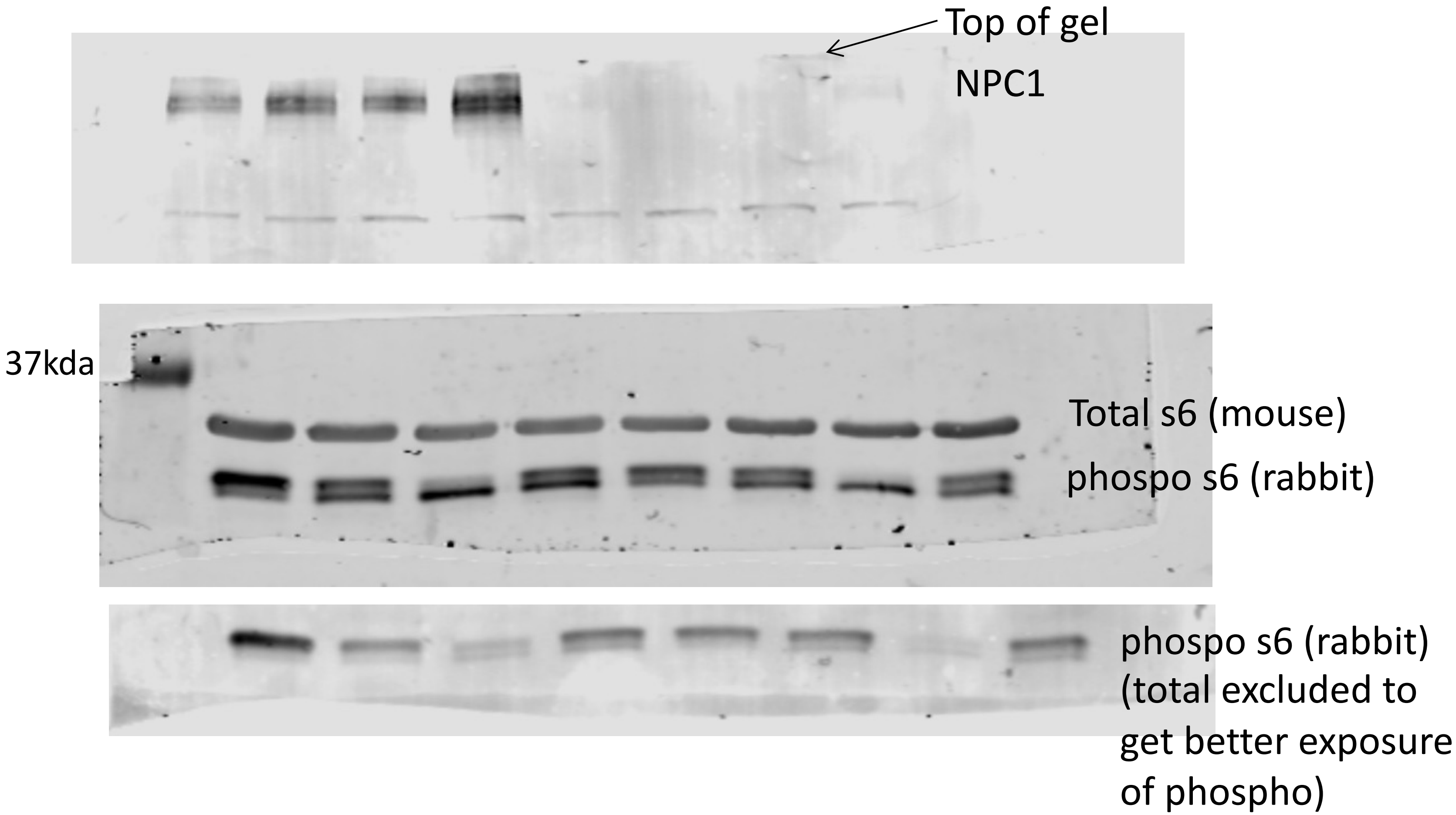

Top of gel
NPC1
37kda
Total s6 (mouse)
phospo s6 (rabbit)
phospo s6 (rabbit)
(total excluded to
get better exposure
of phospho)

Blot 5C (1 of 2)

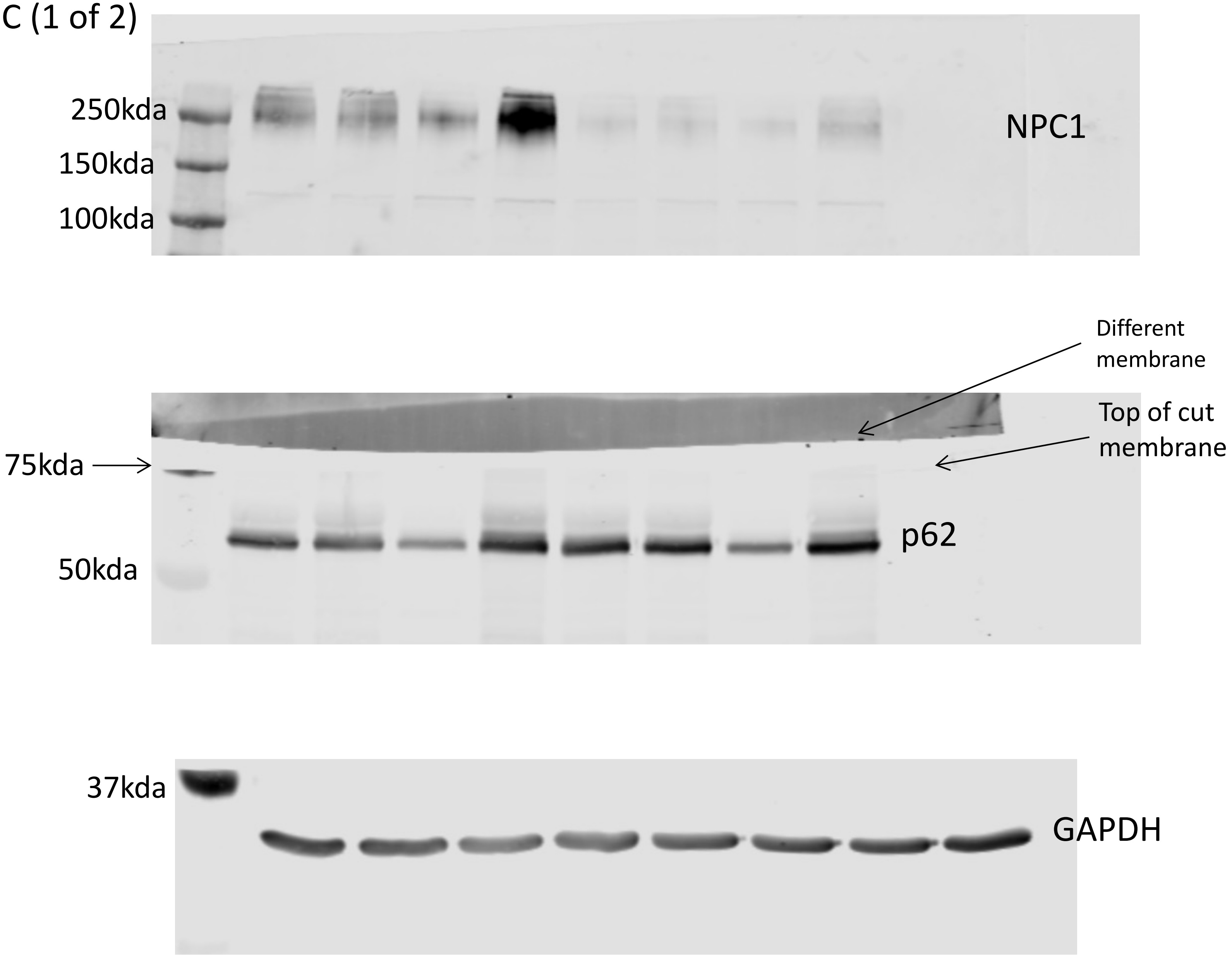

250kda
150kda
100kda
NPC1
Different membrane
Top of cut membrane
75kda
50kda
p62
37kda
GAPDH

Blot 5C (2 of 2)

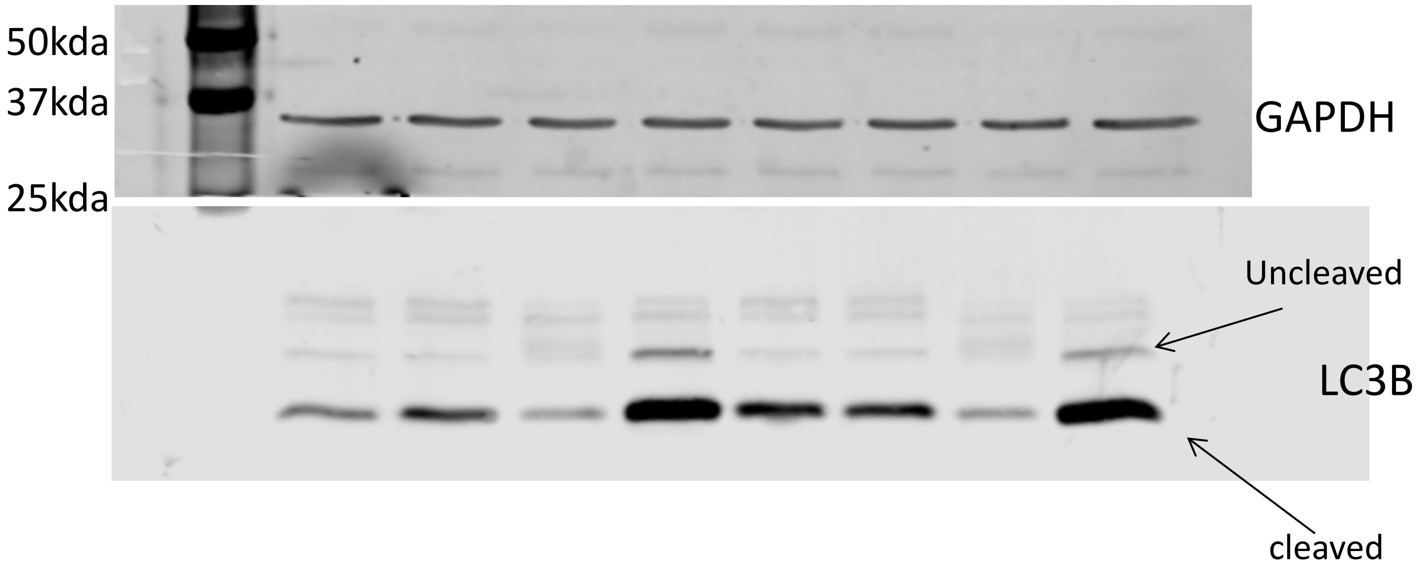

Blot Supp. 1F (Same western as 1B)

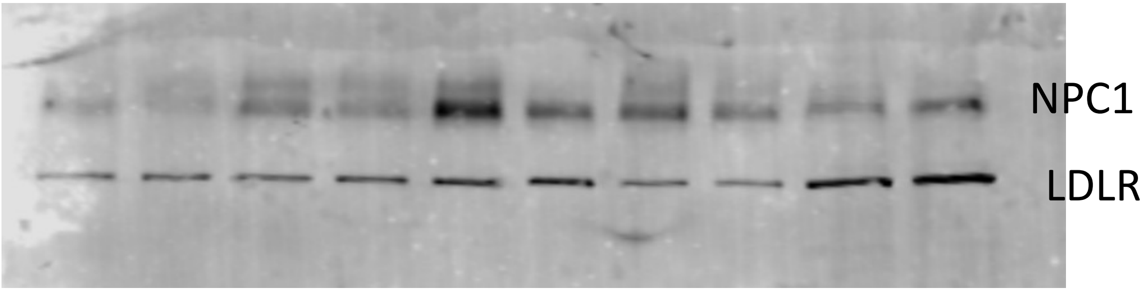

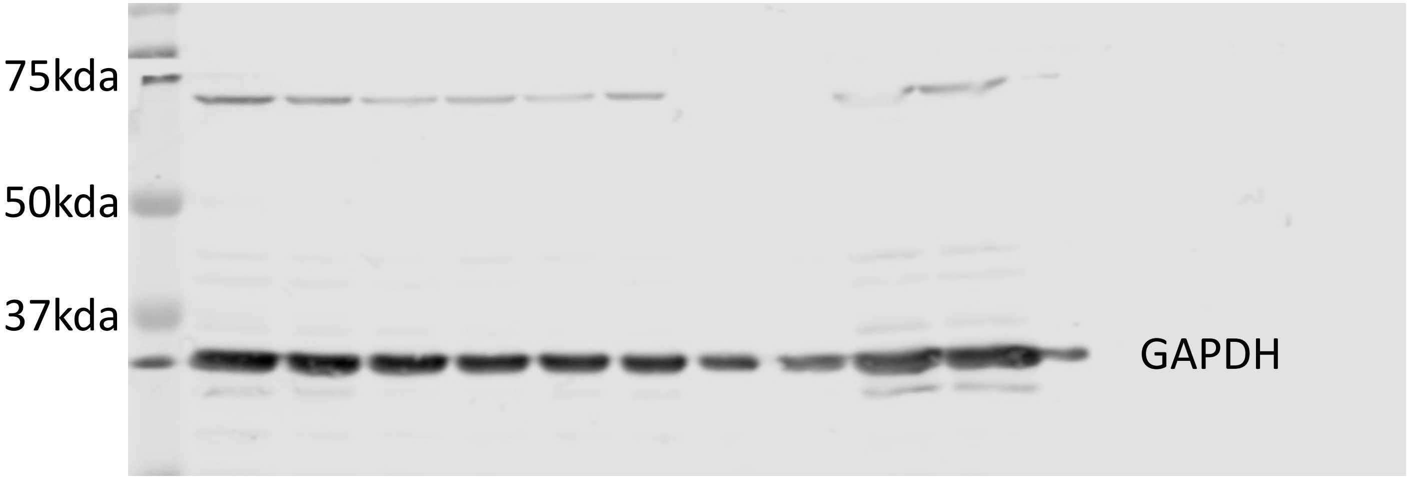

Blot Supp. 3a

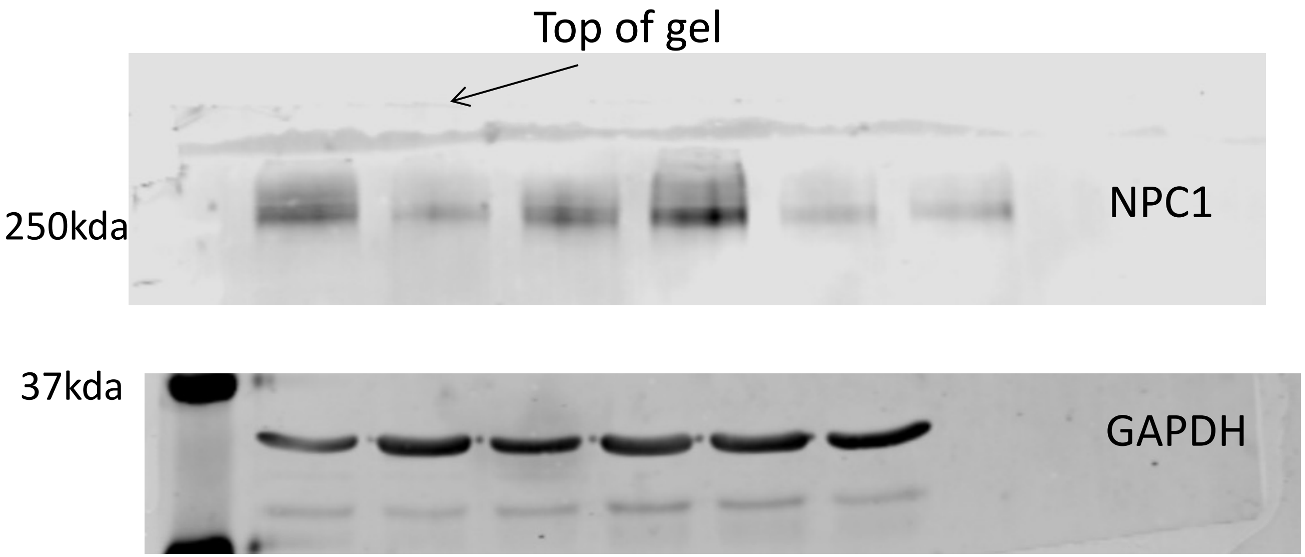

Blot Supp. 4F

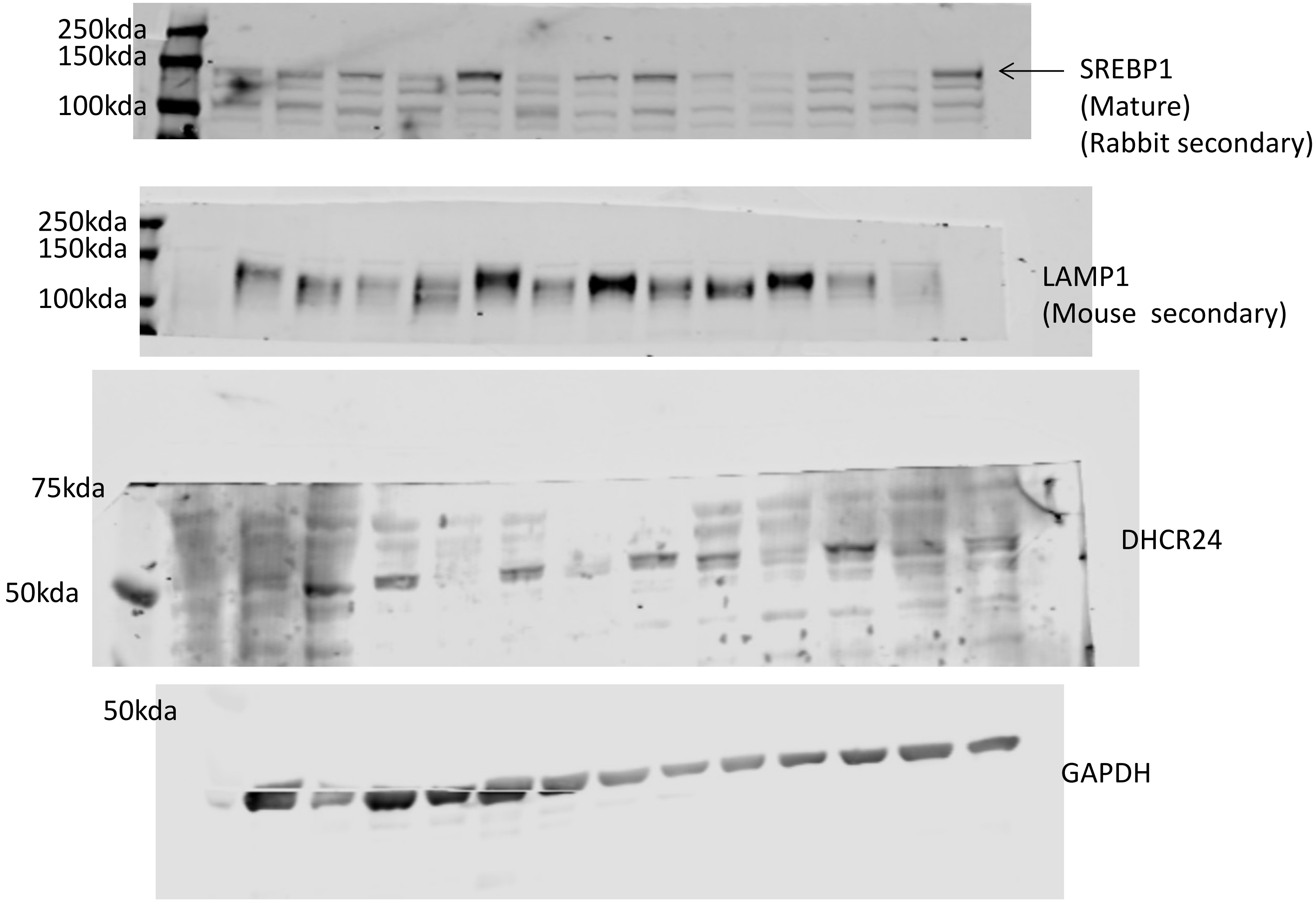
250kda
150kda
100kda
SREBP1
(Mature)
(Rabbit secondary)
250kda
150kda
100kda
LAMP1
(Mouse secondary)
75kda
50kda
DHCR24
50kda
GAPDH

Blot Supp. 8a

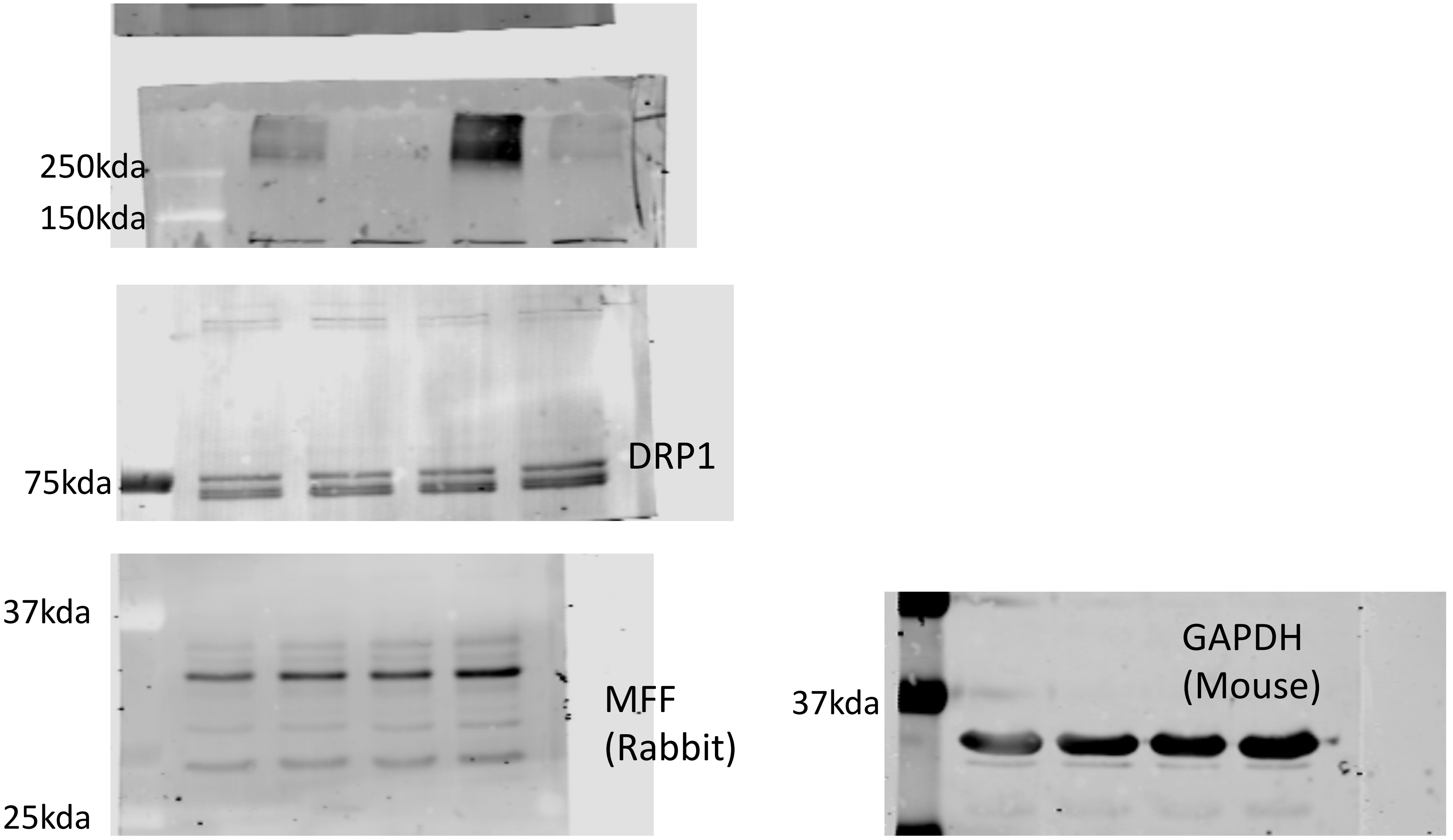
250kda
150kda
75kda
DRP1
37kda
25kda
MFF
(Rabbit)
37kda
GAPDH
(Mouse)
