# Supplementary material for: NPC1 Confers Metabolic Flexibility in Triple Negative Breast Cancer
Source: Cancers (Basel). 2022 Jul 21;14(14):3543. doi: 10.3390/cancers14143543 (PMC9319388; doi:10.3390/cancers14143543)
Supplement: Supplementary file 1 [file cancers-14-03543-s001.zip › Supplemental Data/Final_supplemental figures.pdf]

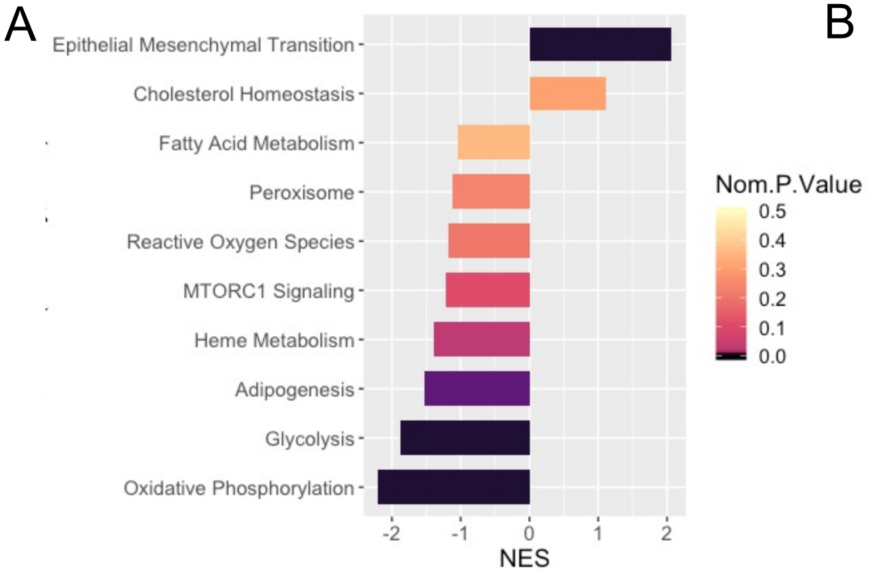

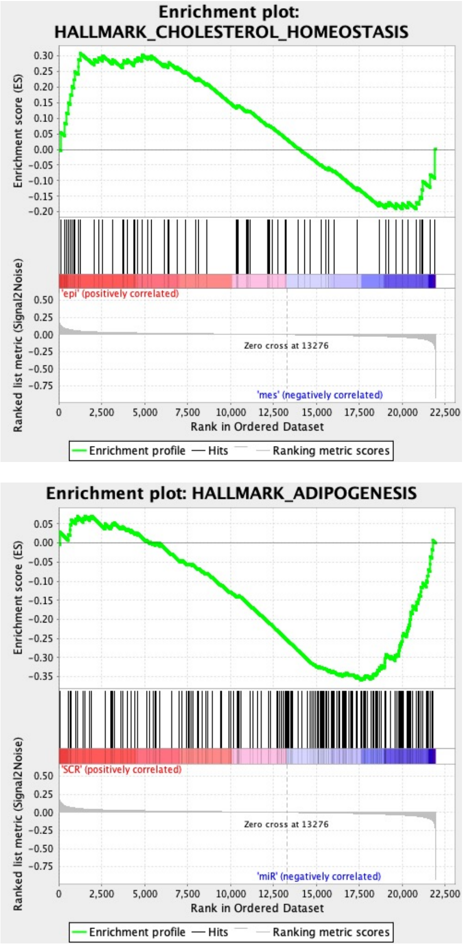

C

GENE	F.C	P-Value
VLDLR	-2.06892	1.22E-05
NPC1	-1.53135	1.28E-05
ENPP2	-1.52916	0.00084
ST3GAL2	-1.39995	0.00054
CAT	-1.30101	0.00178
CMBL	1.30337	0.00164
ANGPTL4	1.34559	0.00131
PEX1	1.35462	5.00E-05
DHRS7	1.43994	0.00102

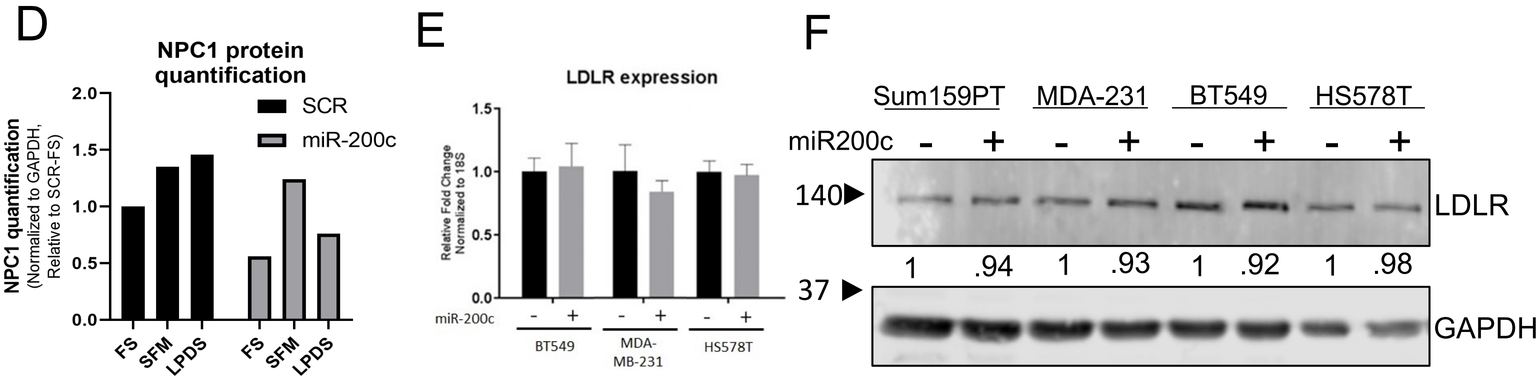

Supplemental Figure S1

A) Metabolic Pathways enriched in previously published gene array (miR-200c -/+ for 72hr in BT549 cells. GSEA analysis using Hallmarks gene set. **B)** GSEA maps for Cholesterol Homeostasis and Adipogenesis genes. **C)** Top hits within cholesterol homeostasis/adipogenesis gene sets (Fold change miR-200c/SCR, P-value student's T Test) **D)** quantification (imageJ) of western blot from figure 1F. **E)** qPCR of *LDLR* expression in TNBC cell lines, 96 hours post miR-200c transfection. **F)** Western of LDLR protein expression, 96 hours post miR-200c transfection.

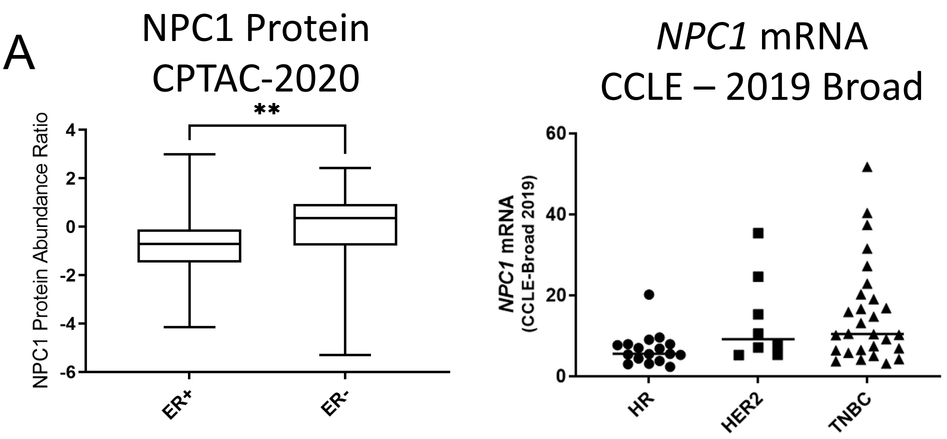

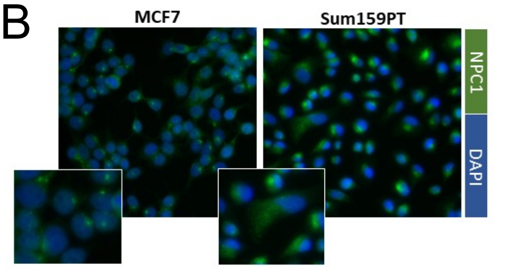

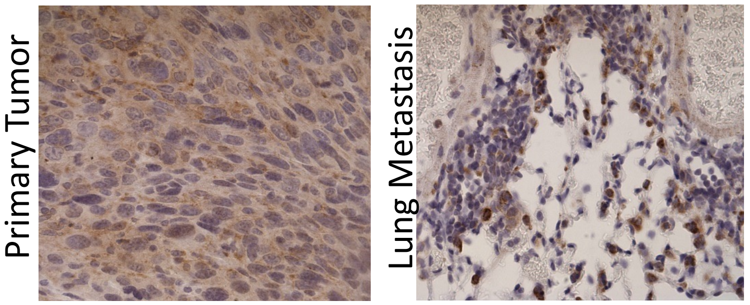

D

Breast Cancer Patient Data

LRP8	0.490	Endocytosis, Cholesterol metabolism
HK3	0.481	Glucose Metabolism
YEATS2	0.480	Histone Modification
MSN	0.467	Cytoskeleton
CASC1	-0.465	Cytoskeleton
RMC1	0.456	Vesicle Trafficking
LYN	0.449	Chemokine signaling; Lipid metabolism
UPP1	0.446	Nucleotide Metabolism
TBC1D9	0.440	Autophagy
FOXA1	0.439	FoxO Signaling

Breast Cancer Cell Lines

TAP2	0.697	Antigen Processing
C7orf57	0.685	Uncharacterized
LRIG3	0.685	EGFR Signaling
UBR4	0.670	Ubiquitin System
CDHR4	0.663	Cell Adhesion
CD109	0.650	CD Molecules
FLRT2	0.650	Cell Adhesion
MYO1E	0.645	Trafficking
ATG9A	0.644	Autophagy
RIO3K	0.643	Kinase Signaling

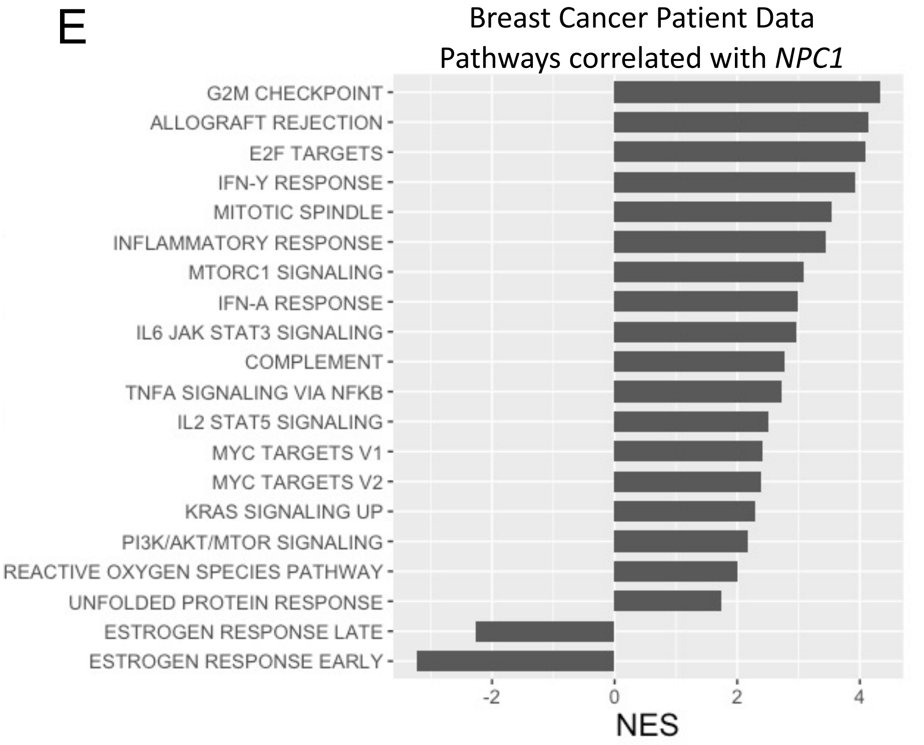

Supplemental Figure S2

A) NPC1 in publicly available datasets: Protein abundance in Breast Cancer-Patient Primary Tumors (Right) and Cancer Cell Line Encyclopedia – Breast Cancer mRNA expression of *NPC1* (Left), accessed through cbioportal. **B)** Immunocytochemistry of NPC1 (green) and DAPI (blue) in MCF7 (ER+) and Sum159PT (TNBC) cells, 10x. **C)** IHC of NPC1 in MMTV-driven mammary tumor (top) and lung metastasis (bottom). **D)** Genes most highly correlated with *NPC1* in 2016 METABRIC patient dataset (*left*) and CCLE-Breast Cancer cell line database (*right*). **E)** Pathway analysis of *NPC1* correlation with genome-wide data from 2016 METABRIC patient breast cancers. Analyzed with GSEA – Pre-ranked, Hallmarks gene sets.

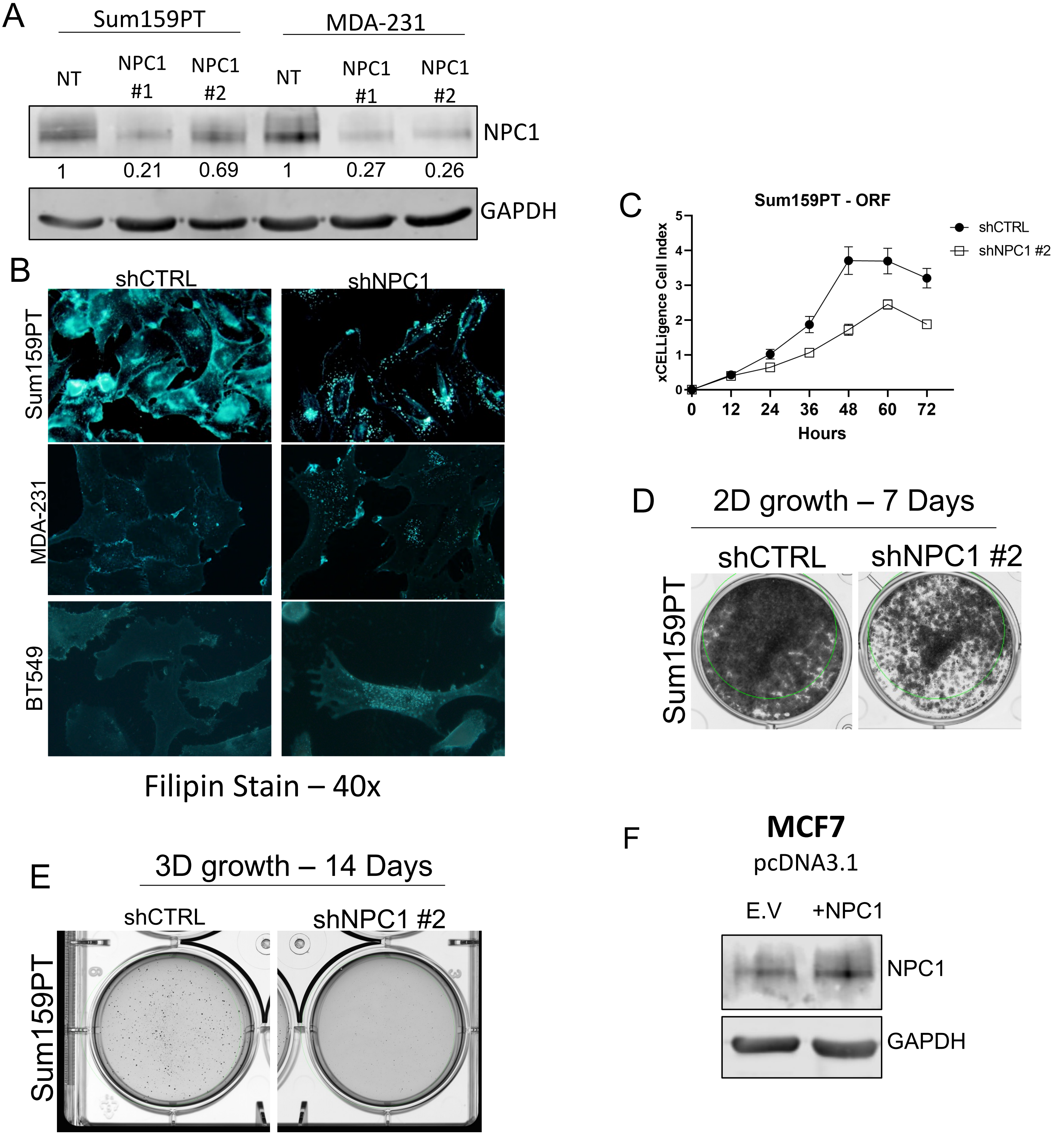

Supplemental Figure S3. **A)** Western blot confirming NPC1 knockdown by two unique shRNAs. **B)** Filipin (neutral cholesterol staining) of stable shNPC1 knockdown versus empty vector cell lines. **C)** xCelligence growth assay of Sum159PT "shRNA #2", shNPC/empty vector over 72 hours. **D)** Representative images of confluency of Sum159PT "shRNA #2" cells after 7 days in 2D culture. 12 well dish with initial seeding density of 3,000 cells/well. **E)** Representative images of colony formation of Sum159PT "shRNA #2" in soft agar (0.5% bottom layer, 0.3% top layer) over 14 days. **F) C**onfirmation of NPC1 overexpression in MCF7 cells used for soft agar and invasion assay, figure 2F-2G.

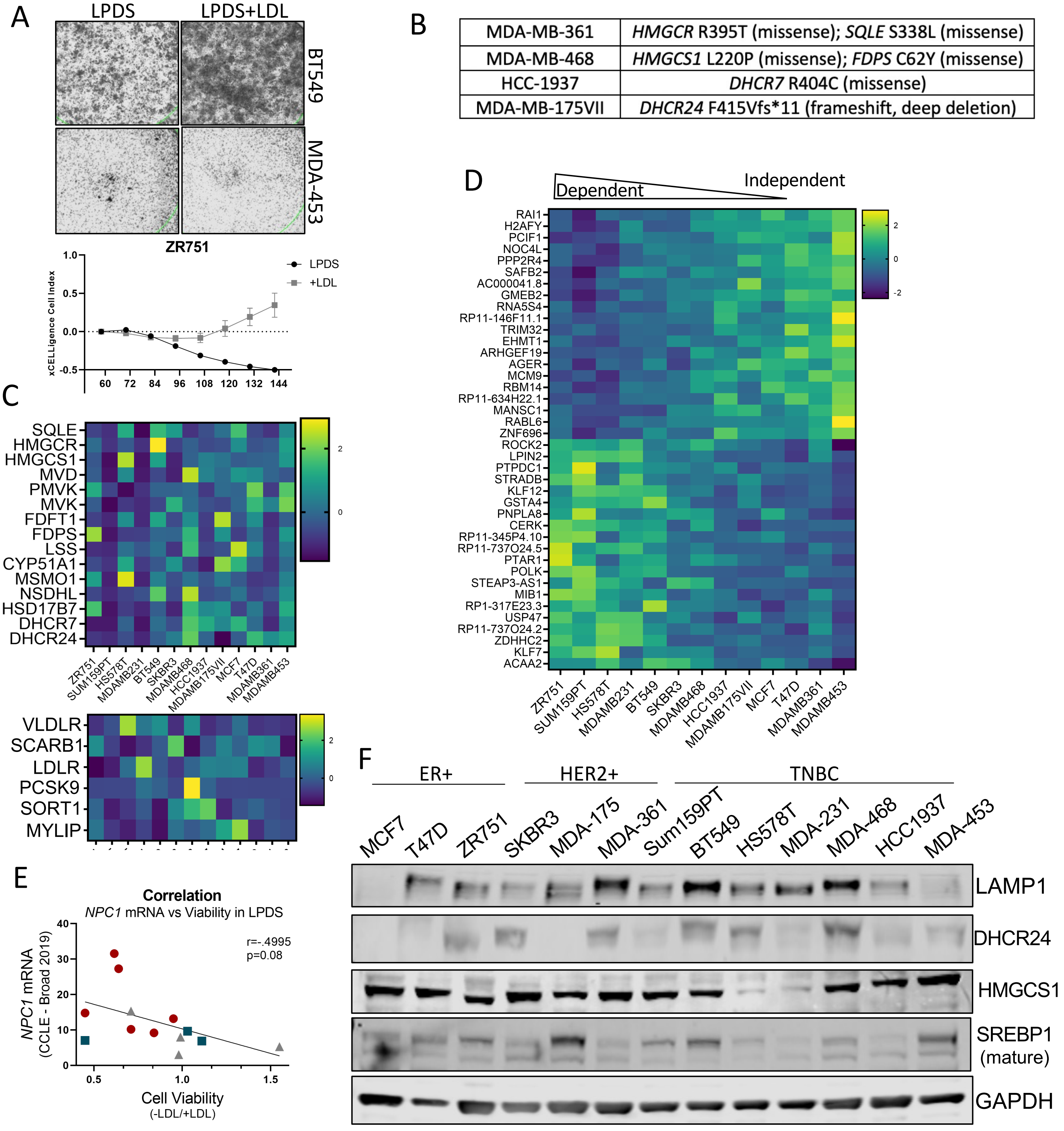

Supplemental Figure S4. **A)** *Top:* growth in LPDS -/+ LDL of MDA-453 (cholesterol independent) and BT549 (cholesterol auxotroph). *Bottom:* Growth over time in ZR75-1 (cholesterol auxotroph) days 2-7, when seeded at equal cell number following 48 hour lipoprotein starvation. **B)** Mutations in cholesterol biosynthesis genes, data obtained from CCLE accessed via cbioportal. **C)** Cholesterol biosynthesis (top) and uptake (bottom) genes in panel of breast cancer cell lines, CCLE via cbioportal.. **D)** Top genes correlated with cholesterol auxotrophy, CCLE accessed via cbioportal. **E)** Correlation of NPC1 mRNA versus cholesterol auxotrophy; mRNA expression obtained from CCLE/cbioportal and compared to viability from figure 3A. **F)** Western of key cholesterol metabolism enzymes in breast cancer cell line panel.

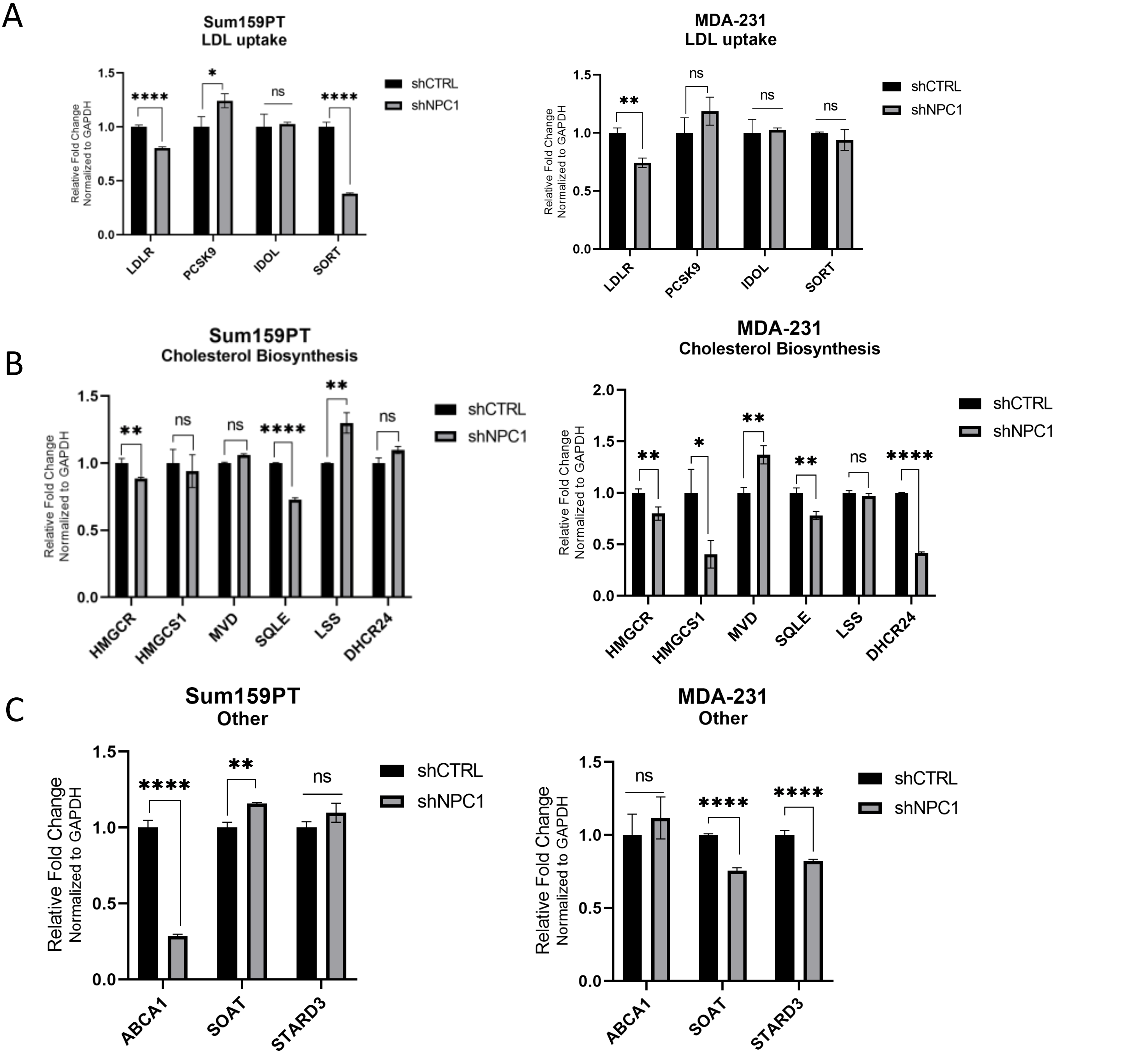

Supplemental Figure S5. qPCR for shRNA#1 cell lines, **A)** genes reportedly involved in cholesterol uptake and LDLR regulation. **B)** Cholesterol biosynthesis genes. **C**) misc. cholesterol-related genes, *ABCA1* (cholesterol efflux), *SOAT1* (cholesterol esterification), and *STARD3* (lysosome-to-mitochondrial cholesterol transporter).

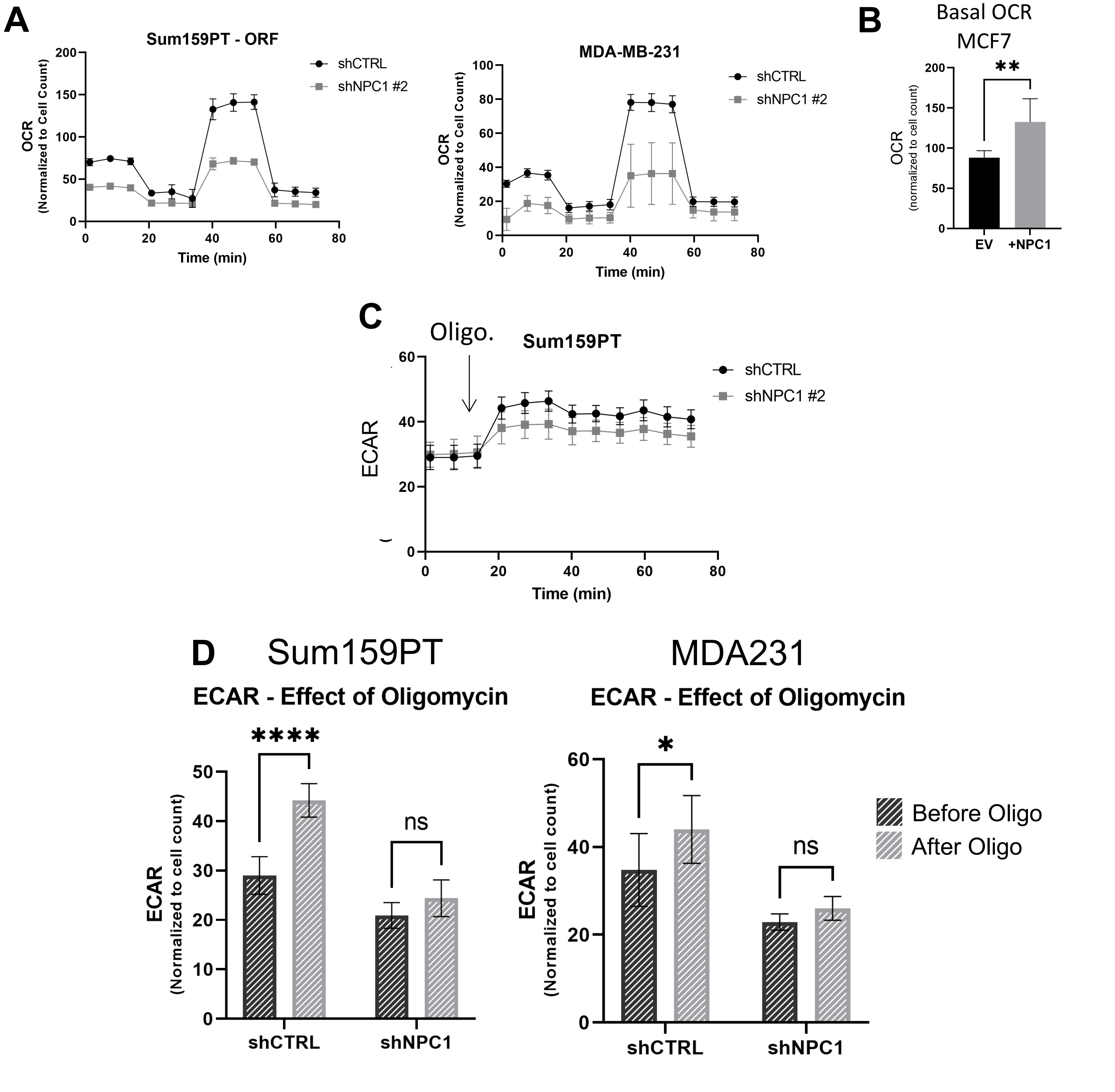

Supplemental Figure S6. **A)** Seahorse mitochondrial stress test in shRNA#2 cell lines. **B)** Quantification of basal respiration (OCR) in MCF7 cells. **C)** ECAR changes in Sum159PT cells, with arrow indicating addition of Oligomycin. **D)** ECAR before and after addition of Oligomycin in Sum159PT and MDA-231 cells, demonstrating glycolytic capacity.

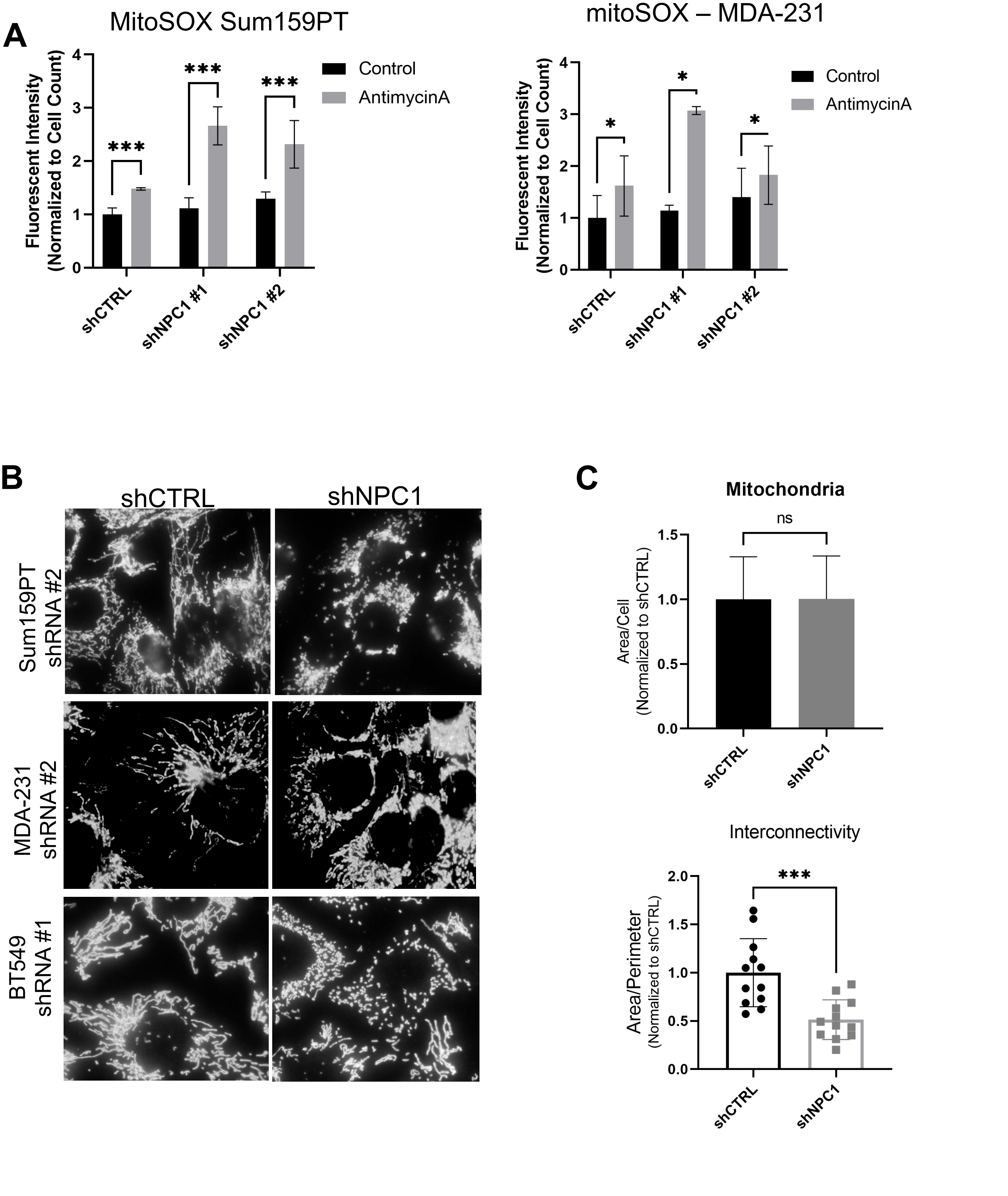

Supplemental Figure S7. **A)** Effect of Antimycin A (used as a positive control) on mitoSOX staining in shCTRL vs. shNPC1 cell lines. **B)** Representative images of mitochondria morphology using additional cell lines/shRNA constructs. **C)** Mitochondrial number (top) and mitochondrial interconnectivity (bottom), based on quantification of confocal microscopy by imagej.

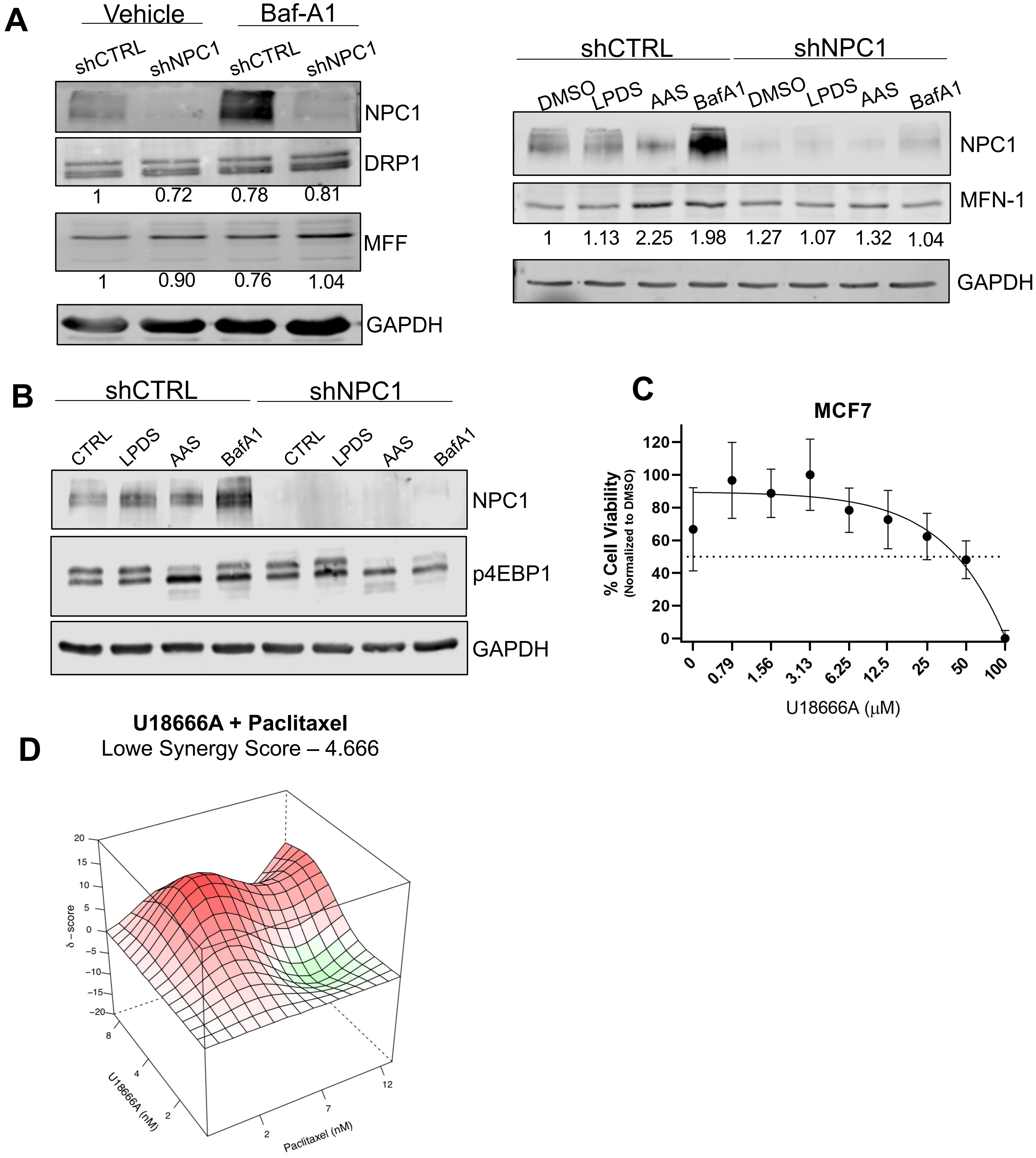

Supplemental Figure S8. **A)** Western blot of various fission/fusion proteins in NPC1+/- cells under various metabolic and lysosome stressors (24 hour treatment. BafA1 5nM). **B)** Western blot of p4EBP1 branch of mTOR signaling under metabolic and lysosome stress. **C)** Dose Response curve of U18666A in MCF7 cells, viability relative to DMSO control over 48 hours, analyzed with Crystal Violet. **D)** Lowe synergy score of U18666A (μM) and Paclitaxel (nM) in BT549 at 48 hours. Calculated using SynergyFinder 2.0.
